# 175. Evaluation of Optimal Urinalysis Criteria for Conditional Urine Culturing

**DOI:** 10.1093/ofid/ofac492.253

**Published:** 2022-12-15

**Authors:** Cecilia Li, Kimberly C Claeys, Daniel J Morgan, K C Coffey, Yeabsera Tadesse

**Affiliations:** University of Maryland School of Pharmacy, Baltimore, Maryland; University of Maryland Baltimore, Baltimore, MD; University of Maryland School of Medicine, Baltimore, Maryland; University of Maryland School of Medicine, Baltimore, Maryland; University of Maryland School of Pharmacy, Baltimore, Maryland

## Abstract

**Background:**

Asymptomatic bacteriuria is a common condition that is often treated unnecessarily with antimicrobials. Conditional urine reflex culturing leverages the negative predictive value of the absence of pyuria, often defined by a minimum urine white blood cell (WBC) count cutoff > 10 cells/hpf. This value has not been extensively validated. This study evaluated parameters from urinalysis (UA), including various WBC cutoffs, and their ability to detect urinary tract infections (UTIs).

**Methods:**

Retrospective cohort study of adult patients with or without urinary catheters with at least one UA between July 2020 to July 2021. Conditional reflex urine culturing with urine WBC cutoff of > 10 cells/hpf was standard practice. Pregnant patients, patients with urologic procedures, and those who were mechanically ventilated at time of UA were excluded. UTIs were defined as definitive, probable, possible, or unlikely (Table 1). Comparisons between definitions and those with definitive/probable versus possible/unlikely UTIs were made using Chi-squared test.

**Table 1.**
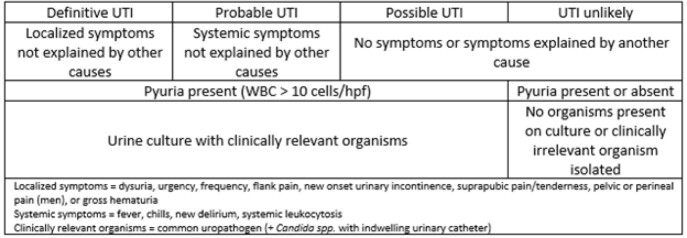
UTI definitions

**Results:**

368 patients were included in the final analysis. 154 (41.8%) were male and mean age was 56.9 ± 17.9 years. 117 (31.8%) had urinary catheters. 296 (80.4%) of UAs were treated with antibiotics which were administered after UA collection 89.2% of the time. The total incidence of definitive, probable, possible, and unlikely UTIs according to WBC count is listed in Table 2. UA parameters including leukocyte esterase positivity and presence of nitrites did not differ when compared between definitive/probable versus possible/unlikely UTIs (94.3% vs. 97.1% and 23.9% vs 26.4% respectively). Differences in leukocyte esterase positivity and presence of nitrites were not seen for non-catheterized patients when compared between definitive/probable versus possible/unlikely UTIs (92.3% vs. 96.5% and 21.2% and 29.3% respectively).

**Table 2.**
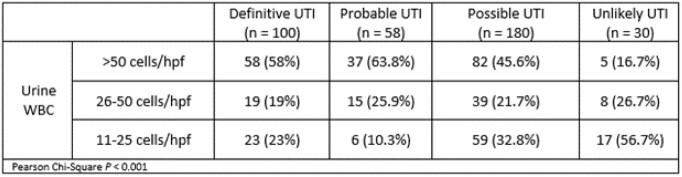
Urine WBC categorization stratified by UTI definition

**Conclusion:**

Urine WBC cutoff > 10 cells/hpf identifies many patients with unlikely UTIs. Current UA parameters do not definitively differentiate UTIs from not in catheterized or non-catheterized patients. Most patients with a positive UA were treated.

**Disclosures:**

**Kimberly C. Claeys, PharmD**, BioFire Diagnostics: Honoraria.

